# Real-World Patterns of *EGFR* Testing and Treatment with Erlotinib for Non-Small Cell Lung Cancer in the United States

**DOI:** 10.1371/journal.pone.0156728

**Published:** 2016-06-13

**Authors:** Lindsey Enewold, Anish Thomas

**Affiliations:** 1 Healthcare Assessment Research Branch, Healthcare Delivery Research Program, Division of Cancer Control and Population Sciences, National Cancer Institute, Bethesda, MD, United States of America; 2 Thoracic and Gastrointestinal Oncology Branch, Center for Cancer Research, National Cancer Institute, Bethesda, MD, United States of America; Peking University People Hospital, CHINA

## Abstract

Despite being the basis of one of the most effective interventions in lung cancer, little is known about the patterns of epidermal growth factor receptor (*EGFR*) mutation testing in the general population. We assessed the frequencies and determinants of *EGFR* testing and erlotinib treatment among a population-based sample. A random sample (n = 1,358) of patients diagnosed in 2010 with histologically-confirmed NSCLC, as reported to the Surveillance Epidemiology and End Results (SEER) program, had their medical records abstracted and treating physicians queried. Logistic regression was used to identify factors associated with *EGFR* testing and erlotinib treatment. Survival was examined using Cox proportional hazards regression. The frequency of *EGFR* testing was 16.8% overall and 22.6% for stage IV adenocarcinoma patients. Given an *EGFR* mutation, 33.6% of all patients and 48.3% of stage IV patients received erlotinib. Among stage IV patients, increased age, Medicaid/no/unknown insurance status, death within 2 months of diagnosis and comorbidity were inversely associated with *EGFR* testing; erlotinib treatment was less likely among smokers and patients with non-adenocarcinomas. *EGFR*-mutation was associated with improved survival, albeit only among stage IV adenocarcinomas. Less than a quarter of NSCLC patients diagnosed in 2010 received *EGFR* testing and less than half of the patients with *EGFR*-mutant stage IV tumors received erlotinib. Significant disparities were observed in *EGFR* mutation testing by health insurance status, comorbidity and age. A national strategy is imperative to ensure that resources and processes are in place to efficiently implement molecular testing of cancer.

## Introduction

Lung cancer is the leading cause of cancer-related mortality in the United States [1]. Non-small cell lung cancer (NSCLC), which constitutes over 80% of all lung cancer, is most often diagnosed at advanced stages and, as such, is largely treated with systemic therapy. Although distinct histological subtypes of NSCLC have been recognized since the 1950s, NSCLC was considered a single disease entity in terms of selection of treatment options until the mid-2000s [2].

It has since been recognized that a subgroup (10–28%) of NSCLCs have mutations in the epidermal growth factor receptor (*EGFR*) gene that predict sensitivity to EGFR tyrosine kinase inhibitors (TKIs), such as gefitinib, erlotinib or afatinib [3–6]. Clinical trials have consistently demonstrated response rates of over 60% with EGFR TKIs in patients with *EGFR*-mutant NSCLC [7–10]. Marked improvements in progression-free survival have also been demonstrated with EGFR TKIs over first-line chemotherapy, albeit with no overall survival benefit potentially due to patient crossover from chemotherapy to EGFR TKI [7–10]. Erlotinib, afatinib and gefitinib are currently approved in the United States for treatment of patients with advanced, *EGFR*-mutant NSCLC tumors.

The observation that tumor genotype affects treatment response has transformed the recommended care of patients with NSCLC; practice guidelines recommend molecular testing of tumor specimens to inform treatment decisions [11–13]. Both the National Comprehensive Cancer Network and American Society of Clinical Oncology guidelines recommended *EGFR* mutation testing for NSCLCs, particularly for advanced, non-squamous tumors [14–15]. However, barriers to molecular testing have been identified [16] and the patterns of *EGFR* mutation testing and erlotinib treatment in the general population have not previously been described.

The primary aims of this study were to estimate the frequency and determinants of *EGFR* mutation testing and erlotinib treatment among a population-based sample of NSCLC patients. All stages of NSCLC were studied, in order to gain broad insights into community practice. A secondary aim was to determine if *EGFR* mutation status and/or receipt of erlotinib were associated with survival. In order to carry out these research aims, we analyzed data from the most recent National Cancer Institute (NCI) Patterns of Care (POC) lung cancer study. The included participants were diagnosed in 2010 with histologically confirmed NSCLC and were ascertained through the Surveillance, Epidemiology and End Results (SEER) program. When these patients were diagnosed, based on the FDA labeling, erlotinib was the preferred EGFR TKI. Although it was not until 2011 that professional guidelines recommended *EGFR* testing, the utility of *EGFR* testing to predict EGFR TKI sensitivity and the benefits of EGFR TKIs in *EGFR*-mutant tumors were well recognized in 2010 [6,7,17–19]. We believe that the findings described herein provide insights into the early dissemination of NSCLC management practices and could inform ongoing efforts to improve uptake of molecular testing in the general population.

## Materials and Methods

### Data source

A NCI POC study was conducted among NSCLC patients who were ascertained through the SEER program. The SEER program consists of multiple population-based registries that collect data, mainly from hospital records, on incident cancer arising within specified geographic regions across the nation. For this POC study, a random sample of eligible NSCLC SEER patients was selected after stratification by registry, sex and race/ethnicity. To increase precision among minority groups, African Americans, Hispanics, Asian Pacific Islanders (APIs) and American Indians/Native Alaskans (AI/NA) were oversampled. Using survey instruments developed for the POC study, trained abstractors at each registry reviewed medical records and treating physicians were queried. Data were collected at least one year post-diagnosis and included demographics, diagnostics, staging, tumor characteristics and treatment. For more details see S1 Methods. Each SEER registry obtained institutional review board approval prior to initiating the POC study.

### Study population

Patients were eligible for the POC study if they were diagnosed in 2010 with invasive, histologically-confirmed, primary NSCLC [International Classification of Diseases for Oncology, 3rd Edition (ICD-O3): C34.X and 8000–8040, 8046–8671 and 8940–8941]. Patients were not eligible if they had a history of cancer, except non-melanoma skin cancer, were simultaneously diagnosed with more than one cancer within 60 days, were diagnosed at autopsy or via death certificate only or were younger than 20 years at diagnosis. For the current analyses, patients with neuroendocrine carcinoma, not otherwise specified (ICD-O3: 8246: n = 25) and patients with unknown tumor stage (n = 22) were also excluded.

### Statistical analysis

To obtain estimates that reflected all eligible NSCLCs diagnosed within the SEER program in 2010, sample weights, defined as the inverse of the sampling proportion for each sampling stratum, were applied. To account for the sample design, all analyses were performed using SAS (version 9.3; SAS Institute Inc., Cary, NC) and SAS-callable SUDAAN (version 10.0.1; Research Triangle Institute, Research Triangle Park, NC).

The weighted percentages of patients who had *EGFR* testing or received erlotinib were calculated among all patients combined and stratified by tumor characteristics (stage, histology and *EGFR* status). Due to the infrequency of *EGFR* testing and administration of erlotinib among patients with non-metastatic disease (stage I-III), subsequent analyses excluded these patients. Among stage IV patients, factors associated with *EGFR* testing and erlotinib treatment were assessed separately. Variables that were associated with either outcome (p<0.10) based on bivariate Chi-square tests were included in a multivariate logistic model and were retained if they remained significant. The weighted percentage of stage IV patients with *EGFR* mutations was also calculated overall and stratified by race/ethnicity and histology. Finally, whether or not *EGFR* mutation status and/or receipt of erlotinib were associated with all-cause mortality was assessed by constructing a multivariate Cox proportional hazards regression model. Follow-up began on the first day of the month of cancer diagnosis; exact diagnosis date was not available. Survival was calculated through the date of death, date of last contact or December 31, 2011, whichever came first. *EGFR* mutations are known to be more common in adenocarcinomas; therefore, sensitivity analyses were also conducted excluding all non-adenocarcinoma patients. All tests were two sided and statistical significance was assessed using an alpha of 0.05.

## Results

### Patient demographics and characteristics

This study included 1,358 NSCLC patients. The mean age was 67.7 years and 74.6% of patients were over 60 years (Table 1). 54.6% were male and 73.8% were non-Hispanic white. Most patients (82.4%) were smokers and had adenocarcinomas (50.5%). According to the American Joint Committee on Cancer 7th edition (AJCC-7), 26.5% were stage I-II, 18.2% were stage III and 55.3% were stage IV.

**Table 1:** Demographics and characteristics among sampled patients diagnosed in 2010 with non-small cell lung cancer, Patterns of Care.

	Total	
	(N^1^ = 1,358)	
Characteristics	N^1^	%^2^
Age at diagnosis		
<50	125	6.3
50–59	287	19.1
60–69	396	30.4
70–79	354	27.8
80+	196	16.4
Mean (standard error)	67.7 (0.5)	
Sex		
Male	690	54.6
Female	668	45.4
Race/Ethnicity		
Non-Hispanic white	369	73.8
Non-Hispanic black	346	11.3
Hispanic	280	6.9
API	302	7.7
AI/AN	61	0.3
Ever Smoker		
No	251	12.0
Yes	1036	82.4
Unknown	71	5.6
Histology		
Adenocarcinoma	754	50.5
Squamous cell	331	27.8
Large cell	56	4.7
Other/not specified	217	17.0
Stage, AJCC7		
I -II	339	26.5
III	255	18.2
IV	764	55.3
Charleson comorbidity index		
0	621	40.7
1+	737	59.3
Died within 2 months of diagnosis		
No	1064	78.8
Yes	294	21.2
Hospital bed size		
< 200 beds, out patient only, unknown	282	25.2
200–299 beds	263	18.0
300–399 beds	311	24.3
400+ beds	502	32.5
Hospital type		
Government, non-federal and federal/unknown	289	20.3
Non-government, not-for-profit	983	73.2
Non-government, for-profit	86	6.5
Approved residency training program		
No/Unknown	572	51.0
Yes	786	49.0

AI/NA: American Indians/Native Alaskans; AJCC7: American Joint Committee on Cancer 7th edition; API: Asian Pacific Islander;

^1^ Unweighted total sample size;

^2^ Weighted percentage

### Frequency of EGFR testing

Overall 16.8% of NSCLC patients underwent *EGFR* testing (Table 2). When stratified by histology, the frequency of *EGFR* testing ranged from 2.7% among large cell tumors to 20.8% among adenocarcinomas. *EGFR* testing tended to be more likely among patients with stage IV disease (all histologies: 19.9%; adenocarcinomas: 22.6%) but variations across stage did not tend to be statistically significant.

**Table 2:** Frequency of *EGFR* testing and receipt of erlotinib among non-small cell lung cancer patients diagnosed in 2010 overall and by tumor stage, Patterns of Care.

	Total	Stage I-II	Stage III	Stage IV	
	%^1^	%^1^	%^1^	%^1^	p^2^
Frequency of *EGFR* testing					
All tumors	16.8	13.9	11.4	19.9	0.08
Adenocarcinoma	20.8	14.4	22.2	22.6	0.23
Squamous cell	12.1	19.6	5.1	10.3	0.22
Large cell	2.7	0	7.1	1.3	0.28*
Other/not specified	16.7	1.4	6.4	26.4	**<0.01**
Frequency of erlotinib treatment					
All tumors	6.3	0.4	6.2	9.2	**<0.01**
*EGFR*-mutant	33.6	0.6	21.8	48.3	**<0.01**
*EGFR*-wild type	5.9	0.0	5.9	8.4	0.44*
*EGFR* status unknown	4.8	0.4	5.2	6.9	**<0.01**

EGFR: epidermal growth factor receptor;

^1^ Weighted percentage;

^2^ Bivariate Chi-square test across all tumor stage or *between stage III and stage IV

### Frequency of erlotinib treatment

Erlotinib was administered to 6.3% of all NSCLC patients, 33.6% of patients with *EGFR*-mutant tumors, 5.9% of patients with *EGFR*-wild type tumors and 4.8% of patients with unknown *EGFR*-mutant status (Table 2). The receipt of erlotinib, increased significantly with stage among all patients (stage I-II: 0.4%; stage III: 6.2%; stage IV: 9.2%; p<0.01), among patients with *EGFR*-mutant tumors (stage I-II: 0.6%; stage III: 21.8%; stage IV: 48.3%; p<0.01) and among patients with tumors of unknown *EGFR* status (stage I-II: 0.4%; stage III: 5.2%; stage IV: 6.9%; p<0.01).

### Determinants of EGFR testing

Among patients with stage IV disease, bivariate analyses indicated that *EGFR* testing was associated with younger age, Hispanic and API heritages, being married, having private/military/other insurance, being a non-smoker, having adenocarcinoma or other/non-specified carcinoma, having no comorbidities and living at least two months after cancer diagnosis (Table 3). Although the likelihood of *EGFR* testing decreased with age, in comparison to patients less than 50 years, multivariate modeling indicated that testing was significantly lower only among patients aged 50–59 [odds ratio (OR): 0.24; 95% confidence interval (CI): 0.08–0.69] and 80 years or older (OR: 0.21; 95% CI: 0.06–0.69). *EGFR* testing was also less likely among patients with any Medicaid or no/unknown insurance compared to those with private/military/other insurance (OR range: 0.15–0.20), among patients with large cell tumors compared to those with adenocarcinomas (OR: 0.04; 95% CI: 0.01–0.23), among patients who had comorbidities (OR: 0.33; 95% CI: 0.16–0.68) and patients who died within two months of their cancer diagnosis (OR: 0.24; 95% CI: 0.08–0.73). Additionally, *EGFR* testing was significantly more likely among Hispanics compared to Non-Hispanic whites (OR: 2.54; 95% CI: 1.28–5.03). Among patients with stage IV adenocarcinomas, *EGFR* testing remained significantly more likely among Hispanics, patients with private/military/other insurance and patients with no comorbidities.

**Table 3:** Factors associated with *EGFR* testing among patients diagnosed in 2010 with stage IV non-small cell lung cancer, Patterns of Care.

	All					Adenocarcinoma				
Characteristic	N^1^	%^2^	p^3^	OR^4^	95% CI	N^1^	%^2^	p^3^	OR^4^	95% CI
Overall	764	19.9				476	22.6			
Age at diagnosis										
<50	74	43.7	0.08	1.00	ref	47	55.3	0.16		
50–59	176	16.6		**0.24**	**0.08–0.69**	126	20.8			
60–69	212	24.0		0.49	0.18–1.33	131	22.9			
70–79	191	16.7		0.39	0.15–1.05	112	19.6			
80+	111	12.3		**0.21**	**0.06–0.69**	60	13.7			
Sex										
Male	420	19.8	0.95			238	25.0	0.51		
Female	344	20.1				238	20.5			
Race/Ethnicity										
Non-Hispanic white	197	19.1	**<0.01**	1.00	ref	116	20.7	**0.02**	1.00	ref
Non-Hispanic black	184	12.5		0.55	0.26–1.15	119	17.3		1.05	0.48–2.30
Hispanic	167	30.1		**2.54**	**1.28–5.03**	95	37.8		**2.88**	**1.30–6.37**
API	185	27.1		1.68	0.78–3.56	129	31.9		1.89	0.82–4.38
AI/AN	31	12.6		0.63	0.12–3.41	17	12.2		0.54	0.06–4.63
Marital Status										
Married/Living as	354	25.6	**0.05**			216	30.2	0.06		
Other	410	15.6				260	18.3			
Median income, $^5^										
>62,000	220	25.0	0.39			142	33.7	**0.05**		
43,000–62,000	248	18.5				170	19.5			
< 43,000	296	16.5				164	15.5			
Insurance										
Private/Military/Other	417	24.6	**<0.01**	1.00	ref	271	29.7	**0.01**	1.00	ref
Medicare only	114	18.4		0.89	0.38–2.06	58	14.1		0.57	0.21–1.51
Any Medicaid	180	8.2		**0.20**	**0.10–0.39**	113	10.0		**0.23**	**0.11–0.45**
None/unknown	53	8.9		**0.15**	**0.04–0.50**	34	11.3		**0.18**	**0.05–0.61**
Ever Smoker										
No	158	36.2	**0.04**			121	39.2	0.06		
Yes	570	16.5				334	17.5			
Unknown	36	33.1				21	40.5			
Histology										
Adenocarcinoma	476	22.6	**<0.01**	1.00	ref					
Squamous cell	123	10.3		0.47	0.13–4.68					
Large cell	37	1.3		**0.04**	**0.01–0.23**					
Other/not specified	128	26.4		1.15	0.51–2.62					
Charleson comorbidity index										
0	367	29.6	**<0.01**	1.00	ref	232	34.6	**<0.01**	1.00	ref
1+	397	11.9		**0.33**	**0.16–0.68**	244	12.2		**0.26**	**0.12–0.58**
Died within 2 months of diagnosis										
No	511	26.2	**<0.01**	1.00	ref	339	28.3	**<0.01**		
Yes	253	6.4		**0.24**	**0.08–0.73**	137	8.3			
Hospital bed size										
< 200 beds, out patient only, unknown	165	20.0	0.72			98	29.2	0.73		
200–299 beds	156	24.1				101	21.8			
300–399 beds	179	15.7				107	20.9			
400+ beds	264	20.8				170	17.8			
Hospital type										
Government, non-federal and federal/unknown	177	26.9	0.33			110	28.0	0.67		
Non-government, not-for-profit	536	18.0				334	21.0			
Non-government, for-profit	51	17.8				32	23.5			
Approved residency training program										
No/Unknown	341	16.0	0.10			204	20.5	0.43		
Yes	423	24.4				272	25.5			

AI/NA: American Indians/Native Alaskans; API: Asian Pacific Islander; CI: confidence interval; EGFR: epidermal growth factor receptor; OR: odds ratio.

^1^ Unweighted total sample size.

^2^ Weighted percentage that had the test (test positive; test negative; test performed, result unknown)

^3^ Bivariate Chi-square test.*When Large cell/Other was combined with Carcinoma, NOS

^4^ Logistic regression model adjusting for all variables that were ≤0.10 during univariate analysis and remained significant ≤0.05 in multivariate analyses.

^5^ Based on aggregate data at the census tract level, Census 2000; tertile cut points based on overall weighted distribution.

### Frequency of EGFR mutations

Overall 30.4% of the patients with stage IV tumors who underwent *EGFR* testing were found to have an *EGFR* mutation (data not shown). When stratified by race/ethnicity, *EGFR* mutations were least common among non-Hispanic whites (21.2%) compared to Non-Hispanic blacks (42.5%), APIs (49.0%) and Hispanic patients (50.1%). *EGFR* mutations were also more common in adenocarcinomas (35.7%) than squamous cell tumors (9.9%) and tumors of other histology (22.4%).

### Determinants of erlotinib treatment

Among patients with stage IV disease, treatment with erlotinib was associated with Hispanic and API heritages, not smoking, having an adenocarcinoma, having an *EGFR* mutation, living at least two months after cancer diagnosis and being treated at a larger hospital (Table 4). Multivariate analysis indicated that erlotinib treatment was significantly less likely among smokers compared to non-smokers (OR: 0.27; 95% CI: 0.12–0.59) and patients with other/not specified NSCLC histologies compared to patients with adenocarcinoma (OR: 0.14; 95% CI: 0.04–0.54) and more likely among patients with *EGFR* mutations (OR: 9.90; 95% CI: 3.04–32.24). Among patients with stage IV adenocarcinomas, receipt of erlotinib remained significantly lower among smokers and higher among patients with *EGFR* mutations. Residence in a higher median income area was also significantly associated with receipt of erlotinib. Of patients who received erlotinib, 87.0% of patients with *EGFR*-mutant tumors started erlotinib as their first-line therapy compared to 36.2% of patients with *EGFR* wild-type tumors (data not shown).

**Table 4:** Factors associated with receipt of Erlotinib among patients diagnosed in 2010 with stage IV non-small cell lung cancer, Patterns of Care.

	All					Adenocarcinoma				
Characteristic	N^1^	%^2^	p^3^	OR^4^	95% CI	N^1^	%^2^	p^3^	OR^4^	95% CI
Overall	764	9.2				476	12.4			
Age at diagnosis										
<50	74	16.4	0.41			47	23.5	0.26		
50–59	176	5.7				126	7.0			
60–69	212	10.4				131	13.3			
70–79	191	7.4				112	9.3			
80+	111	11.6				60	19.1			
Sex										
Male	420	8.3	0.53			238	11.2	0.63		
Female	344	10.2				238	13.5			
Race/Ethnicity										
Non-Hispanic white	197	6.7	**<0.01**			116	9.7	0.06		
Non-Hispanic black	184	9.6				119	11.3			
Hispanic	167	16.2				95	23.2			
API	185	23.1				129	25.5			
AI/AN	31	8.9				17	11.6			
Marital Status										
Married/Living as	354	9.7	0.74			216	12.8	0.88		
Other	410	8.7				260	12.2			
Median income, $^5^										
>62,000	220	14.7	0.11			142	24.7	**0.02**	1.00	ref
43,000–62,000	248	7.0				170	7.1		**0.30**	**0.11–0.83**
< 43,000	296	6.0				164	6.7		**0.34**	**0.13–0.91**
Percentage with a high school education^5^										
>89%	331	7.1	0.15			206	9.2	0.06		
77–89%	221	5.9				147	7.0			
<77%	212	13.9				129	20.9			
Insurance										
Private/Military/Other	417	11.5	0.07			271	16.4	0.06		
Medicare only	114	3.7				58	5.1			
Any Medicaid	180	6.9				113	5.7			
None/unknown	53	7.9				34	10.3			
Ever Smoker										
No	158	23.8	**<0.01**	1.00	ref	121	24.8	**<0.01**	1.00	ref
Yes	570	5.9		**0.27**	**0.12–0.59**	334	7.5		**0.31**	**0.13–0.73**
Unknown	36	24.8		1.93	0.26–14.53	21	44.0		2.95	0.43–20.09
Histology										
Adenocarcinoma	476	12.4	**<0.01**	1.00	ref					
Squamous cell	123	5.9		0.70	0.19–2.52					
Large cell	37	8.7		1.08	0.15–8.09					
Other/not specified	128	2.0		**0.14**	**0.04–0.54**					
*EGFR* mutation										
Negative	84	8.4	**<0.01**	1.00	ref	63	10.3	**0.01**	1.00	ref
Positive	68	48.3		**9.90**	**3.04–32.24**	54	58.3		**14.46**	**3.59–58.22**
Unknown	612	6.9		0.72	0.27–1.93	359	9.0		0.89	0.24–3.48
Charleson comorbidity index										
0	367	11.9	0.13			232	16.1	0.18		
1+	397	6.9				244	9.1			
Died within 2 months of diagnosis										
No	511	11.5	**0.03**			339	14.2	0.27		
Yes	253	4.1				137	7.8			
Hospital bed size										
< 200 beds, out patient only, unknown	165	8.6	**0.04**			98	8.7	**0.05**		
200–299 beds	156	13.4				101	18.4			
300–399 beds	179	4.3				107	6.4			
400+ beds	264	11.1				170	16.3			
Hospital type										
Government, non-federal and federal/unknown	177	7.6	0.48			110	11.9	0.73		
Non-government, not-for-profit	536	8.1				334	11.1			
Non-government, for-profit	51	25.5				32	26.4			
Approved residency training program										
No/Unknown	341	9.0	0.90			204	10.5	0.30		
Yes	423	9.4				272	15.0			

AI/NA: American Indians/Native Alaskans; API: Asian Pacific Islander; CI: confidence interval; OR: odds ratio.

^1^ Unweighted total sample size.

^2^ Weighted percentage that received Erlotinib.

^3^ Bivariate Chi-square test.

^4^ Logistic regression model adjusting for all variables that were ≤0.10 during univariate analysis and remained significant ≤0.05 in multivariate analyses.

^5^ Based on aggregate data at the census tract level, Census 2000; tertile cut points based on overall weighted distribution.

### Survival

Among patients with stage IV disease, *EGFR*-mutant tumors and treatment with erlotinib were both associated with better survival during bivariate analyses (Table 5). However, in multivariate analyses, neither retained survival significance. Among patients with stage IV adenocarcinomas, patients with *EGFR*-mutant tumors had a better survival [Hazard Ratio (HR): 0.43; 95% CI: 0.24–0.76]; again, receipt of erlotinib was not associated with survival.

**Table 5:** Association between *EGFR*-mutant status and Erlotinib treatment with all-cause survival among patients diagnosed in 2010 with stage IV non-small cell lung cancer, Patterns of Care.

	All					Adenocarcinoma				
	N^1^	(%)^2^	p^3^	HR^4^	95% CI	N^1^	(%)^2^	p^3^	HR^4^	95% CI
*EGFR* mutation										
Negative	84	66.3	**<0.01**	1.00	ref	63	73.0	**<0.01**	1.00	ref
Positive	68	45.6		0.64	0.35–1.18	54	32.1		**0.43**	**0.24–0.76**
Unknown	612	86.0		**1.59**	**1.18–2.14**	365	81.6		1.06	0.71–1.58
Erlotinib										
No, unknown	662	83.6	**0.01**	1.00	ref	401	79.7	**0.04**	1.00	ref
Yes	102	63.0		0.69	0.47–1.02	81	60.2		0.69	0.47–1.03

EGFR: epidermal growth factor receptor; CI: confidence interval; HR: hazard ratio.

^1^ Unweighted total sample size.

^2^ Weighted percentage of patients who had died as of December 31, 2011.

^3^ Bivariate Chi-square test by vital status as of December 31, 2011.

^4^ Hazard ratio from Cox proportional hazard model adjusting for age, sex, race/ethnicity, marital status, residential income level, insurance status, ever smoking status, Charlson comorbidity index, *EGFR* status, receipt of Erlotinib, surgery, radiotherapy, other systemic therapy, and hospital characteristics (bed size, classification, residency program)

## Discussion

This study establishes the general population-based patterns of *EGFR* mutation testing and treatment with erlotinib for NSCLC in the United States in 2010. An estimated 16.8% of all newly diagnosed NSCLC patients underwent *EGFR* mutation testing. Among patients with stage IV tumors, *EGFR* testing varied significantly by age, insurance and comorbidity level. Furthermore, an estimated 33.6% of NSCLC patients with *EGFR*-mutant tumors received erlotinib, which was also administered to 5.9% of NSCLC patients with *EGFR*-wild type tumors. Among patients with stage IV tumors, erlotinib treatment was less likely among smokers and patients with non-adenocarcinomas. *EGFR*-mutation was associated with improved survival, albeit only among stage IV adenocarcinomas.

The reason why a large proportion of patients with stage IV disease in the current study were not assessed for *EGFR* mutations is likely multifactorial. For example, assay costs and issues related to tissue acquisition and turnaround time may have contributed to the low testing rate [20,21]. Additionally, and maybe more importantly, professional guidelines did not recommend routine testing for *EGFR* mutations until 2011 [11–13]. However, given that the benefit of *EGFR*-directed therapy in selected patients was recognized well before professional societies formally recommended testing, it was still surprising that less than a quarter of the patients underwent *EGFR* testing in 2010.

More concerning is our finding of significant disparities in *EGFR* mutation testing. In addition to observing variations by health insurance status, comorbidity and older age were associated with significantly lower *EGFR* mutation testing rates. Although aggregate residential income level was not associated with *EGFR* testing rates, it cannot be ruled out that the association with insurance status may at least partially be due to confounded by unmeasured variations in patient-level variables (e.g., income and education). The observed variations in *EGFR* testing rates by insurance status may also reflect the fact that professional guidelines, which can impact insurance coverage policy, had yet to recommend routine *EGFR* testing by 2010. It is worth noting however that previous POC analyses have indicated that patients with Medicaid or Medicare-only are often under treated [22]; thus, our results may extend these findings to the realm of molecular testing. Given the manageable toxicity profile and higher efficacy compared with chemotherapy, EGFR TKIs are recommended in patients with tumors harboring *EGFR* sensitizing mutations regardless of their performance status [23]. Sustained, clinically relevant improvements in quality of life have been observed in patients with *EGFR-*mutant tumors after EGFR TKIs treatment compared to chemotherapy [7]. Our data indicates that in the general practice, comorbid conditions and limited life expectancy, both surrogates for poor performance status, were significant negative determinants of *EGFR* mutation testing. Although the progression-free survival benefits associated with erlotinib have not been found to vary by age, greater toxicities have been observed among older patients [24]. Therefore, the anticipation of greater toxicities with erlotinib among older patients may explain why older age was associated with a lower likelihood of *EGFR* testing.

Disparities were also observed for the receipt of erlotinib related to smoking status and possibly median residential income level. Although further studies are needed to confirm these findings, it is possible that clinicians are less inclined to administer erlotinib to smokers because smoking increases the metabolic clearance of erlotinib and, thereby, diminishes the effectiveness [25]. The finding that lower income patients were less likely to receive erlotinib may be a true indication of a cost barrier. However, this finding should be interpreted cautiously because individual income level was not available.

The incidence of *EGFR* mutation varies by race/ethnicity. Previous studies have estimated that 15–20% of white and 50–55% of API NSCLC patients have *EGFR*-mutant tumors [26–29]. The frequency of *EGFR* mutations among African American and Hispanic NSCLC patients is less clear. Possibly due to small sample sizes and heterogeneity across studies, the frequency of *EGFR*-mutant tumors among African American NSCLC patients has ranged between 2–20% [26–30]. Only one study has assessed the frequency of *EGFR*-mutant NSCLC in Hispanic patients (15%) [31]. In contrast to these previous studies, the frequency of *EGFR*-mutant tumors in this study was assessed among patients selected on the basis of clinico-pathologic characteristics (e.g., patients with adenocarcinomas and/or non-smokers were more likely to have an *EGFR* test). Thus, the observed frequencies in the current study demonstrate selective testing tends to enrich the frequency of *EGFR*-mutant tumors, particularly among non-Hispanic black and Hispanic patients. Higher than expected frequencies of *EGFR*-mutant tumors among non-Hispanic black and Hispanic patients, may be largely due to racial/ethnic variation in smoking and histology. For example, Hispanic patients were less likely to be smokers than NHW (71.1% vs. 85.4%, respectively, p<0.01) and Hispanic and non-Hispanic black patients were more likely to have had adenocarcinomas than NHW patients (56.7%, 57.5% and 48.5%, respectively p≤0.14; data not shown).

Albeit only among patients with stage IV adenocarcinomas, we found that *EGFR*-mutant tumors were associated with better survival. This finding is consistent with previous reports in patients with advanced NSCLC, which suggest that *EGFR* mutation status by itself is a favorable prognostic marker [32,33]. Erlotinib was not independently associated with survival. However, due to the high frequency of patients in whom *EGFR* testing was not done, the small number of patients with *EGFR* mutations and the observational nature of this study, which makes it prone to confounding by indication, these results should be interpreted with caution.

To our knowledge only a limited number of prior studies have addressed the question of adoption of *EGFR* mutation testing in general practice. Based on retrospective data from the US Oncology Network data, Pan et al. estimated that 15.2% of patients with stage IV NSCLC underwent *EGFR* testing and that 50.0% of patients with stage IV *EGFR*-mutant tumors received erlotinib [34]. Our results which are based on a larger, population-based sample are consistent with these findings.

The current study also provides data on *EGFR* mutation testing and erlotinib use among patients with early-stage NSCLC, a group for which current guidelines do not recommend testing or treatment [13,26]. Interestingly, 13.9% of NSCLC patients with stage I-II disease received *EGFR* mutation testing. Notwithstanding the relatively high frequency of *EGFR* mutation testing in this population, a very low number of the patients received erlotinib. The role of adjuvant erlotinib in NSCLC remains under investigation (NCT02194738).

The strengths of this study include the population-based data, oversampled minority groups, and physician verified treatment. This study had several limitations. We were not able to assess all factors that may have influenced the decisions to have *EGFR* testing or treatment with erlotinib. For example, tumors that were classified as non-adenocarcinoma may have had an adenocarcinoma component, which may partially explain the higher than expected frequency of *EGFR* mutations among the non-adenocarcinoma categories. Additionally, we were not able to assess how testing and subsequent treatment were impacted by inadequate tissue samples and variable laboratory turnaround times. Variations by specific mutation were also not assessable because this information was not recorded. Additionally, small sample size precluded the ability to identify factors associated with *EGFR* testing and receipt of erlotinib among patients with earlier stage tumors and non-adenocarcinomas. Small sample size may also have impacted the observed mutation rate among racial subgroups. Finally, because NSCLC has not been selected as a POC study cancer site since 2010, it was not possible to assess more recent clinical practices. It is likely that *EGFR* testing frequency has increased in the recent years with it being recommended by professional societies [11,12]. Despite the limitations, we were able to examine *EGFR* testing and erlotinib use among NSCLC patients that were representative of those seen in the general clinical practice.

In conclusion, targeted therapy in molecularly selected patients is transforming lung cancer treatment. Although the current testing rates are likely substantially higher than rates reported here, the results from the current study indicate patterns of early dissemination of *EGFR* mutation testing and erlotinib treatment. The complexity of testing and treatment for lung cancer patients will likely increase as additional targets and therapies are identified. A national strategy is imperative to ensure that resources and processes are in place to more widely implement molecular testing.

## Supporting Information

S1 Methods Description of analysis of individual variables. (DOC)
