# Supplementary material for: Real-World Patterns of EGFR Testing and Treatment with Erlotinib for Non-Small Cell Lung Cancer in the United States
Source: PLoS One. 2016 Jun 13;11(6):e0156728. doi: 10.1371/journal.pone.0156728 (PMC4905679; doi:10.1371/journal.pone.0156728)
Supplement: S1 Methods — (DOC) [file pone.0156728.s001.doc]

**SUPPLEMENTARY METHODS**

**Description of analysis of individual variables**

*EGFR* mutation status, regardless of the method used for identification or the specific mutation, was recorded as: test not performed/no mention; test positive; test negative; test performed, result unknown; and unknown if test performed. *EGFR* testing was considered performed if recorded as test positive; test negative; or test performed, result unknown. *EGFR* mutation status was then grouped as test positive, test negative or unknown (test performed, result unknown; and unknown if test performed).

The receipt of specific systemic therapies, including erlotinib, and the date(s) of initiation were determined based on physician verification. (None of the patients in the current study received gefitinib.) After coding the receipt of individual chemotherapy, a combined any chemotherapy (yes/no) variable was created. The receipt of surgery and radiotherapy were determined based on hospital medical record abstraction and were also coded as binary (yes/no) variables.

Tumor stage was classified according to the American Joint Committee on Cancer 7^th^ edition [1]. Histologic categories adenocarcinoma, squamous, large cell and other/not specified carcinomas were defined according to the International Association of Cancer Research as used in Cancer Incidence in Five Continents, Volume IX [2].

All comorbid conditions listed in the medical record at the hospital where the most definitive treatment was received were recorded and centrally coded at NCI. The Charlson comorbidity index score was then calculated, excluding lung cancer [3]. Smoking status was determined based on hospital medical record abstraction.

Demographic characteristics (age, sex, race/ethnicity and marital status) were determined based on hospital medical records; if ethnicity was unavailable the North American Association of Central Cancer Registries Hispanic Identification Algorithm was used to assign ethnicity [4]. Patient-level insurance status (private/military/other, Medicare only, any Medicaid,

None/unknown) was determined according to the hospital medical record at the time of the most definitive procedure. Patient-level data on income are not collected by the SEER program. Instead median family income (“income”) in the census tract where the patient lived at cancer diagnosis was used as a proxy, based on the 2000 Census data. Income was then categorized into three groups based on the tertiles for the weighted distribution of the analytic dataset.

The environment within which health care is delivered may impact clinical practice patterns. Therefore, hospital bed size, type (government/non-government, not-for-profit/for-profit) and presence of an approved residency training program where the patient had their most definitive treatment were assessed, according to data from the American Hospital Association Annual Survey of Hospitals [5].

**References**
